# A newly identified interaction between nucleolar NPM1/B23 and the HTLV-I basic leucine zipper factor in HTLV-1 infected cells

**DOI:** 10.3389/fmicb.2022.988944

**Published:** 2022-12-01

**Authors:** Zhenlong Liu, Émilie Larocque, Yongli Xie, Yong Xiao, Guy Lemay, Jean-Marie Peloponese, Jean-Michel Mesnard, Éric Rassart, Rongtuan Lin, Shuang Zhou, Yiming Zeng, Hongzhi Gao, Shan Cen, Benoit Barbeau

**Affiliations:** ^1^Département de chimie, Université du Québec à Montréal, Montréal, QC, Canada; ^2^Centre d’excellence en recherche sur les maladies orphelines-Fondation Courtois, Université du Québec à Montréal, Montréal, QC, Canada; ^3^Lady Davis Institute, Jewish General Hospital & Department of Medicine, McGill University, Montreal, QC, Canada; ^4^Département de microbiologie et immunologie, Université de Montréal, Montréal, QC, Canada; ^5^Institute of Medicinal Biotechnology, Chinese Academy of Medical Sciences, Beijing, China; ^6^Institut de Recherche en Infectiologie de Montpellier (IRIM), CNRS, Université Montpellier, Montpellier, France; ^7^Département des sciences biologiques, Université du Québec à Montréal, Montréal, QC, Canada; ^8^Neurosurgery Department, 2nd Affiliated Hospital of Fujian Medical University, Quanzhou, Fujian, China

**Keywords:** HTLV-1, ATLL, HBZ, NPM1/B23, APH-2, nucleolus, RNA binding, cell transformation

## Abstract

Human T-cell leukemia virus type 1 is the causative agent of HTLV-1-associated myelopathy/tropical spastic paraparesis and adult T-cell leukemia-lymphoma (ATL). The HTLV-1 basic leucine zipper factor (HBZ) has been associated to the cancer-inducing properties of this virus, although the exact mechanism is unknown. In this study, we identified nucleophosmin (NPM1/B23) as a new interaction partner of HBZ. We show that sHBZ and the less abundant uHBZ isoform interact with nucleolar NPM1/B23 in infected cells and HTLV-1 positive patient cells, unlike equivalent antisense proteins of related non-leukemogenic HTLV-2, −3 and-4 viruses. We further demonstrate that sHBZ association to NPM1/B23 is sensitive to RNase. Interestingly, sHBZ was shown to interact with its own RNA. Through siRNA and overexpression experiments, we further provide evidence that NPM1/B23 acts negatively on viral gene expression with potential impact on cell transformation. Our results hence provide a new insight over HBZ-binding partners in relation to cellular localization and potential function on cell proliferation and should lead to a better understanding of the link between HBZ and ATL development.

## Introduction

It is estimated that 5 to 10 million individuals are infected by HTLV-1 worldwide, although most infected individuals are healthy asymptomatic carriers (AC; Gessain and Cassar, 2012). Among them, 5% of infected individuals will eventually develop Adult T-cell leukemia-lymphoma (ATL; Mortreux et al., 2003; Verdonck et al., 2007; Yasunaga and Matsuoka, 2007), while 1–2% will progress toward the neurological disorder, HTLV-1-associated myelopathy/tropical spastic paraparesis (HAM/TSP; Bangham et al., 2015). Currently, there are very limited therapeutic treatments available for these diseases. Interestingly, other members of the HTLV family, known as HTLV-2, -3, and-4, have not been associated with any human malignancies despite their strong similarity in genomic structure and viral genes (Calattini et al., 2005; Feuer and Green, 2005; Wolfe et al., 2005; Duong et al., 2008).

Several studies have been devoted to the mechanistic understanding of ATL. One of the HTLV-1 gene associated to ATL is HTLV-I basic leucine zipper factor (*hbz*), which is encoded by the antisense strand of the HTLV-1 genome (Gaudray et al., 2002; Satou et al., 2006; Barbeau et al., 2013; Barbeau and Mesnard, 2015). Two isoforms (sHBZ and uHBZ) have been identified and are expressed from spliced and unspliced transcripts, respectively (Cavanagh et al., 2006; Murata et al., 2006; Satou et al., 2006; Usui et al., 2008). The *shbz* isoform is the most abundant and is expressed at least at four-time higher levels than *uhbz* in HTLV-1-infected cells (Murata et al., 2006). Localization studies have further demonstrated differences between uHBZ and sHBZ: although both HBZ isoforms target the nucleus through three nuclear localization signals (NLS), most studies have demonstrated that sHBZ also localizes to the nucleolus, while uHBZ displays a non-nucleolar distribution (Hivin et al., 2005; Murata et al., 2006). HBZ also contains a Leucine zipper (LZ) motif, which allows it to interact with several other LZ motif-bearing transcription factors (Gaudray et al., 2002; Ma et al., 2016). These LZ-dependent interactions as well as additional interactions with other segments of the proteins are considered important for the different functions attributed to sHBZ, such as maintenance of viral latency through inhibition of the HTLV-1 transactivator Tax and induced proliferation of infected malignant cells (Ma et al., 2016).

The association of sHBZ with ATL development has been supported by reports showing that *shbz* transcripts are detected in most ATL cells from patients and have been shown to be more abundant in ATL patients versus asymptomatic carriers and HAM/TSP patients, mostly correlating with proviral load (Murata et al., 2006; Satou et al., 2006; Saito et al., 2009). Further support for the contribution of sHBZ in ATL development has been associated to proliferation studies showing that sHBZ and possibly its transcript could be essential players in the proliferation of ATL cells (Satou et al., 2006; Arnold et al., 2008; Satou et al., 2011; Mitobe et al., 2015). Reports have also underscored the oncogenic potential of sHBZ in transgenic mice and in NOD/SCID mice engrafted with HTLV-1-transformed T cells (Arnold et al., 2008; Satou et al., 2011; Zhao et al., 2014). Several mechanisms have been proposed to account for the capacity of HBZ to transform cells (Kuhlmann et al., 2007; Kawatsuki et al., 2016; Kinosada et al., 2017). These studies have focused on the sHBZ isoform, since previous results had suggested that the unspliced HBZ isoform might not be oncogenic, as it is unable of inducing IL-2-independent proliferation in IL-2-dependent cell lines (Satou et al., 2006; Yoshida et al., 2008). Of further importance, the equivalent antisense protein encoded by HTLV-2, APH-2 (Antisense protein of human T-cell leukemia virus type 2) is also frequently expressed in primary cells from HTLV-2-infected individuals (Halin et al., 2009), but does not induce IL-2-independent growth in IL-2-dependent T cell lines and localizes to the nucleus, but not to the nucleolus, similarly to uHBZ (Douceron et al., 2012).

The nucleolus is a dynamic cellular structure and, in addition to its essential role in ribosome biogenesis, is involved in many biological processes, such as transcription, cell cycle control, genomic stability, cellular aging and viral infection (Pederson, 2011). The two most abundant nucleolar phosphoproteins, B23 (also known as nucleophosmin, NPM1) and C23 (also termed nucleolin) are implicated in these processes (Box et al., 2016; Scott and Oeffinger, 2016; Jia et al., 2017). Although mainly localized to the nucleolus, these proteins are known to shuttle constantly and freely between the nucleus and the cytoplasm (Borer et al., 1989). Important links between hematological cancers and NPM1/B23 have been highlighted in previous years. Indeed, mutations in the *NPM1* gene has been observed in Anaplastic large-cell lymphoma (ALCL), Acute promyelocytic leukemia (APL) and Acute myeloid leukemia (Pandolfi, 1996; Yoneda-Kato et al., 1996; Grisendi et al., 2006; Kunchala et al., 2017).

Importantly, NPM1/B23 plays functional roles in different stages of viral replication including nuclear import, viral genome transcription and assembly, as well as final particle formation by directly interacting with various viral proteins in different cellular compartments during relevant infections, as exemplified with Chikungunya virus (CHIKV), HIV, HBV, HCV, HDV, HPV and HSV-1 (Nouri et al., 2015; Rawlinson and Moseley, 2015; Abraham et al., 2017). HIV-1 can induce the acetylation of NPM1/B23, which plays a critical role in nuclear localization of Tat and Tat-mediated transcriptional activation of the integrated provirus (Gadad et al., 2011). Furthermore, HIV-1 Rev. and HTLV-1 Rex are known to be targeted to cell nucleoli to enhance viral replication (Greco, 2009; Arizala et al., 2018). In fact, a NPM1/B23 inhibitor, avrainvillamide-analog-3, has in been repurposed as a broad antiviral replication inhibitor (Lobaina and Perera, 2019; Bonnet et al., 2022).

Interestingly, sHBZ interacts with many cellular partners (Ma et al., 2016), although nucleolar proteins have never been previously investigated. Based on the nucleolar localization of sHBZ and the implication of nucleolar proteins in transformation-related processes, we hypothesized that these proteins could be interacting with sHBZ and might be implicated in properties attributed to the pathogenesis of sHBZ. We report herein that NPM1/B23 is a new potential HBZ-interacting partner, an association which is not apparent with antisense proteins from other HTLV viruses. Basic regions of sHBZ and the RNA-binding domain of NPM1/B23 were found to mediate this interaction, while RNA interfered with sHBZ-NPM1/B23 complex formation. Association of sHBZ with its own transcripts was further demonstrated. We finally showed that, by silencing approach and overexpression studies, this association between NPM1/B23 and sHBZ could promote cell transformation. Our results further suggest that the association of HBZ with NPM1/B23 could partly modulate HBZ expression and could account for the role of sHBZ in HTLV-1-associated ATL.

## Results

### Increased expression of *NPM1*/B23 in PBMC from ATL patients and association with sHBZ in transfected cells

**Figure 1:**
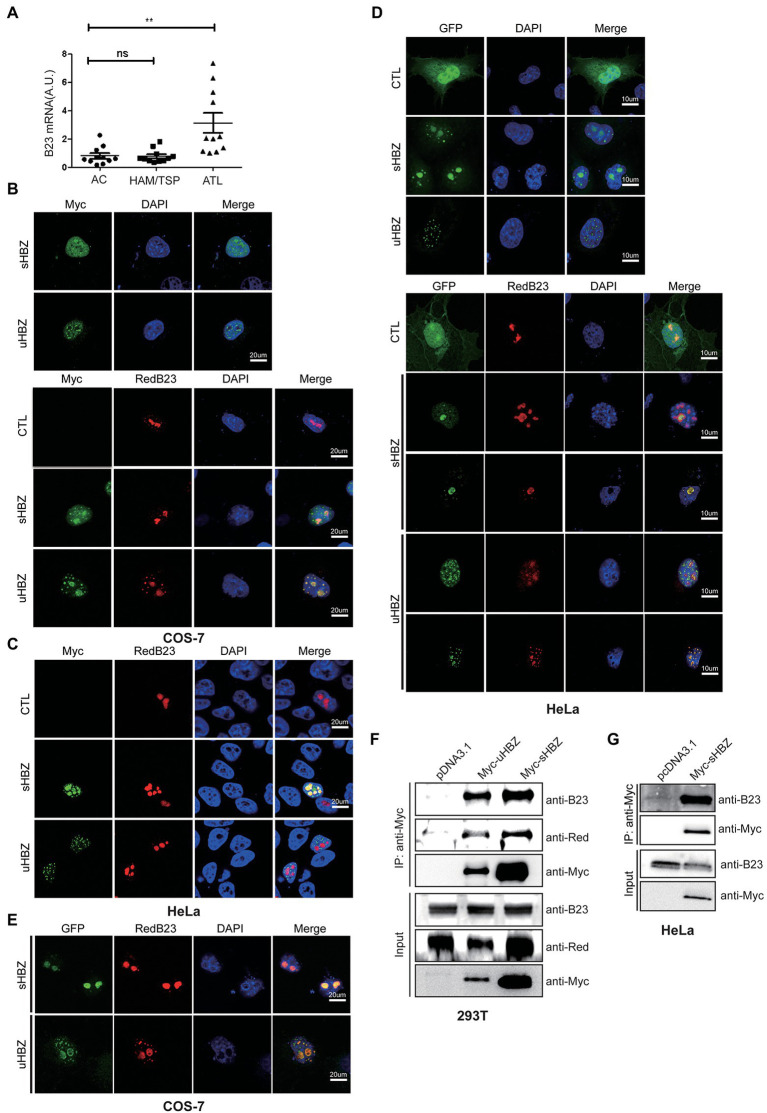
Expression of *NPM1*/B23 in PBMC from infected individuals and association with sHBZ in transfected cells. **(A)** RNA from PBMCs of asymptomatic individuals (AC) or HAM/TSP and ATL patients of Caribbean origin was analyzed by qRT-PCR for *NPM1*/B23 expression (**p < 0.01). COS-7 **(B)** and HeLa **(C)** cells were co-transfected with expression vectors for Myc-tagged sHBZ or uHBZ (vs. pcDNA3.1) alone or in the presence of pDsRed-B23. Fixed cells were incubated with anti-Myc antibodies followed by goat anti-mouse IgG coupled to Alexa Fluor 488 and stained with DAPI. Cells were then analyzed by confocal microscopy. HeLa **(D)** and COS-7 **(E)** cells were co-transfected with pEGFP-C1-sHBZ, pEGFP-C1-uHBZ or pEGFP-C1 (CTL) and pDsRed-B23. Cells were fixed and stained with DAPI prior to confocal analyses. **(F)** Expression vectors of Myc-tagged sHBZ or uHBZ (vs. pcDNA3.1) and pDsRed-B23 were co-transfected in 293 T. Cells lysates were immunoprecipitated with an anti-Myc antibody. Immunoprecipitates and cell extracts were analyzed by Western blot using anti-B23, anti-DsRed or anti-Myc antibodies. **(G)** Expression vectors of Myc-tagged sHBZ or uHBZ (vs. pcDNA3.1) were transfected in HeLa cells. Cells lysates were immunoprecipitated with an anti-Myc antibody. Immunoprecipitates and cell extracts were analyzed by Western blot using anti-B23 or anti-Myc antibodies. The experiments were repeated three times.

Previous studies have demonstrated that sHBZ is distributed in the form of nuclear speckles and that it also localized to the nucleolus (Murata et al., 2006). The uHBZ isoform shows a similar nuclear pattern, although it does not predominantly localize to the nucleoli (Hivin et al., 2005). NPM1/B23 being a major nucleolar protein implicated in hematological malignancies (Grisendi et al., 2006), we hypothesized that a functional association between HBZ and NPM1/B23 could exist. To address this possibility, we first assessed NPM1 expression in HTLV-1-infected individuals, using PBMCs isolated from HTLV-1-infected asymptomatic individuals and from patients suffering from ATL or HAM/TSP. Following qRT-PCR analyses (Figure 1A), we observed that *NPM1* expression was more pronounced in samples from ATL patients, while no significant increase was noted in HAM/TSP patients (Figure 1A). Of note, no correlation could be demonstrated between proviral load of individuals/patients and *NPM1* expression (data not shown).

Since the above results showed an increase in *NPM1* expression in sHBZ-expressing PBMC from ATL patients, we next sought to further examine a potential association between both nucleolar-located proteins. We initially co-transfected Myc-tagged sHBZ or uHBZ with a DsRed-fused B23 expression vector in COS-7 and HeLa cells (Figures 1B,C). As expected, we first observed that, although both HBZ isoforms were distributed as nuclear speckles, sHBZ also showed important localization to the nucleolus, while uHBZ was more non-nucleolar (Figure 1B). Furthermore, strong co-localization between sHBZ and DsRed-B23 was apparent, especially at the nucleolar region, while important co-localization could be visualized for uHBZ although slightly different pattern of localization was noted at the nucleoli. To further confirm these co-localization data, similar co-transfection experiments of GFP-HBZ and DsRed-B23 expression vectors were performed in HeLa cells (Figure 1D). Higher co-localization was more clearly noted for the sHBZ isoform with a predominant nucleolar localization, while co-localization was generally demonstrated for uHBZ, despite being less prominent than for sHBZ. Similar results were obtained in transfected COS-7 cells (Figure 1E; Supplementary Figure S1). Interestingly, in these cells, we noted that sHBZ was more prominent in the nucleoli when NPM1/B23 was overexpressed, while a higher number of uHBZ-expressing cells showed apparent relocalization of uHBZ to the nucleolar NPM1/B23-positive region (Figure 1E).

To clearly determine if the different HBZ isoforms could associate with NPM1/B23, extracts from 293 T cells transfected with Myc-tagged HBZ and DsRed-B23 expression vectors were immunoprecipitated with anti-Myc antibodies and analysed by Western blot. As depicted in Figure 1F, sHBZ strongly associated to DsRed-B23 as well as to NPM1/B23. Although levels of uHBZ were lower than sHBZ levels, due to its inherent instability, uHBZ nonetheless showed a specific signal for NPM1/B23. Extracts from HeLa cells transfected with Myc-tagged HBZ also revealed association between sHBZ and NPM1/B23, when immunoprecipitated samples were analysed by Western blot (Figure 1G).

These data hence demonstrated that both isoforms of HBZ associated to NPM1/B23. Although nucleolar localization of HBZ was not strictly required, it seemed to favor the interaction between both proteins.

### Association between sHBZ and NPM1/B23 in transfected T cell lines and HTLV-1-infected cells

**Figure 2:**
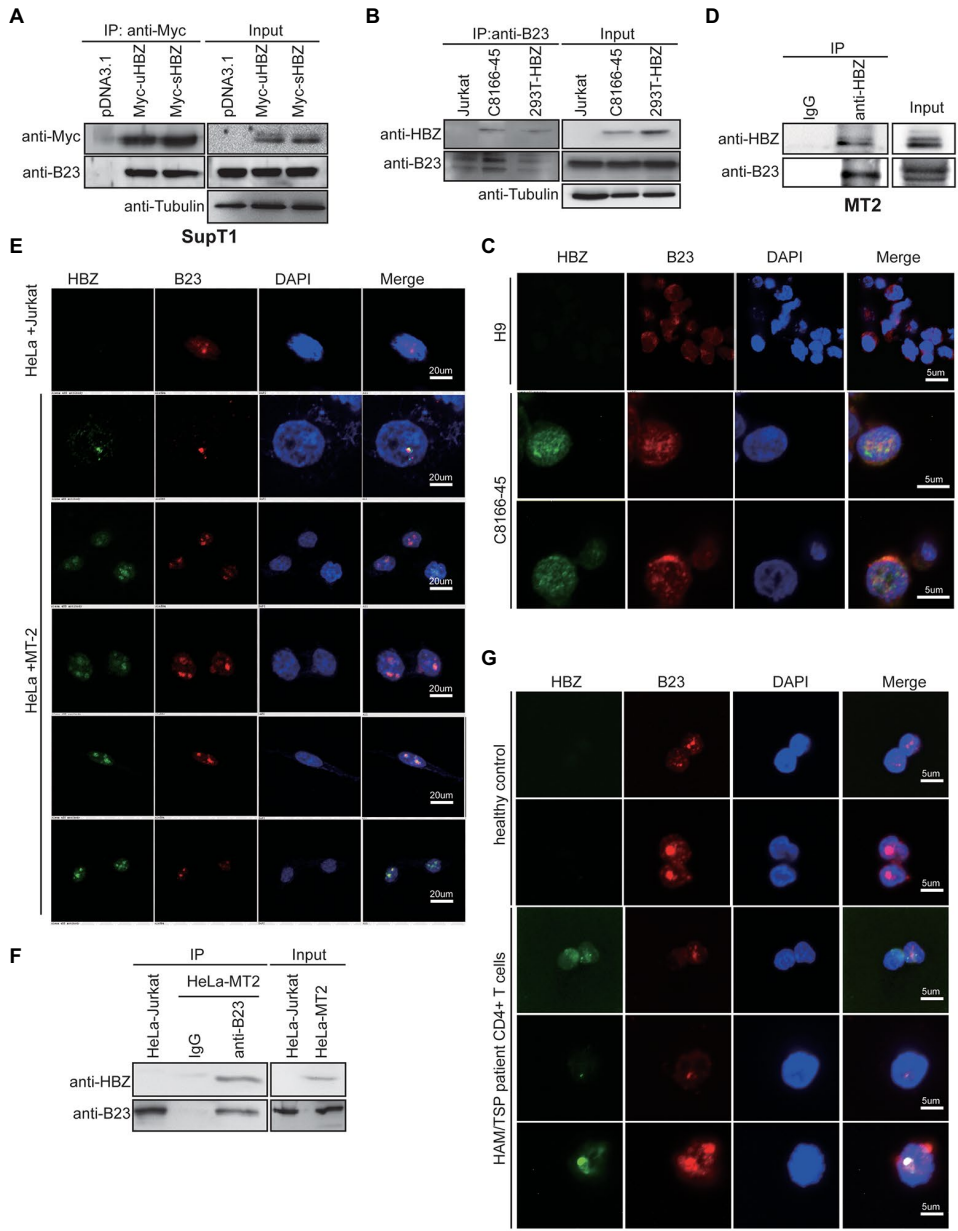
sHBZ interacts with NPM1/B23 in transfected and infected T cells. **(A)** SupT1 cells were transfected with the Myc-tagged sHBZ or uHBZ expression vector (vs. empty vector pcDNA3.1) and extracts were next immunoprecipitated with anti-Myc antibodies. Western blot analyses were conducted with anti-Myc, anti-B23 or anti-tubulin antibodies. **(B)** Extracts from Jurkat, C8166-45 and 293 T cells transfected with the Myc-tagged sHBZ expression vector were immunoprecipitated with anti-B23 antibodies. Immunoprecipitates and total cellular extracts were subsequently analyzed by Western blot using anti-HBZ, anti-B23 or anti-tubulin antibodies. **(C)** C8166-45 and uninfected H9 cells were analyzed for NPM1/B23 and HBZ localization by confocal microscopy. **(D)** MT2 cell extracts were immunoprecipitated with anti-HBZ antibodies (vs. non-specific IgG) and total and immunoprecipitated extracts were analyzed by Western blot using anti-B23 or anti-HBZ antibodies. **(E)** HeLa cells were co-cultured with Jurkat or MT2 cells for 5 days and HeLa cells were analyzed by confocal microscopy for HBZ and NPM1/B23. **(F)** Extracts from HeLa cells co-cultured with Jurkat or MT2 were immunoprecipitated with anti-B23 or non-specific IgG antibodies and analysis of immunoprecipitated and total extracts was performed by Western blot using anti-HBZ or anti-B23 antibodies. **(G)** CD4+ T cells from an uninfected individual and an HTLV-1-infected patient were analyzed by confocal microscopy for NPM1/B23 and HBZ. For confocal microscopy experiments **(C,E,G)**, cells were incubated with anti-HBZ and anti-B23 antibodies, followed by goat anti-mouse IgG coupled to Alexa Fluor 488 and goat anti-rabbit IgG coupled to Alexa Fluor 594, respectively and final DAPI staining. These experiments were repeated three times.

To further validate the association between sHBZ and NPM1/B23, T cell lines were first analysed in transfection experiments. Hence, SupT1 T cells were transfected with Myc-tagged uHBZ and sHBZ expression vectors and subsequently analysed by co-immunoprecipitation (Figure 2A). Both HBZ isoforms indeed formed a complex with NPM1/B23 in this T cell line.

To address this interaction with more relevant expression levels of sHBZ, two chronically infected T cell lines (C8166-45 and MT2), were tested. These experiments were important, as previous studies had suggested that HBZ did not reside in the nucleolar compartment when analysed in non-overexpressing HBZ ATL cell lines and in PBMCs from a patient diagnosed with severe acute ATL (Raval et al., 2015). Extracts from C8166-45, sHBZ-expressing 293 T and uninfected Jurkat cells were immunoprecipitated with anti-B23 antibodies (Figure 2B). Following Western blot analyses, HBZ-specific signals were detected in both immunoprecipitated 293 T and C8166-45 extracts. No similar signals were obtained in these experiments with non-HBZ-expressing Jurkat cells or when extracts were immunoprecipitated with control non-specific IgG antibodies (Figure 2B; Supplementary Figure S2). Confocal microscopy analyses of C8166-45 cells demonstrated co-localization of HBZ and NPM1/B23, while specificity of the HBZ signal was confirmed by the absence of any signals in uninfected H9 cells (Figure 2C). The interaction between HBZ and endogenous NPM1/B23 was also confirmed by co-immunoprecipitation experiments in chronically infected MT2 cells (Figure 2D).

We further tested the HBZ-NPM1/B23 interaction in freshly infected cells. Using a previously described protocol (Liu et al., 2008), HeLa cells were co-cultured with MT2 cells, which were then removed by thorough washing. As shown in Figure 2E, HBZ was specifically detected in infected HeLa cells. When infected HeLa cells were further analysed, a clear co-localization between NPM1/B23 and sHBZ signals could again be demonstrated. Importantly, following immunoprecipitation with anti-B23 antibodies, HBZ was detected in infected HeLa cells and not in uninfected cells. Furthermore, immunoprecipitation of infected HeLa cell extract with a control IgG did not lead to the HBZ-specific signal (Figure 2F). HBZ and NPM1/B23 co-localization was also confirmed in freshly isolated CD4+ T cells from a HAM/TSP patient by confocal microscopy (Figure 2G).

These results hence clearly demonstrated that sHBZ co-localized and was associated to NPM1/B23 in HTLV-1-infected cells and CD4+ T cells from a HAM/TSP patient and that this interaction appeared most prominent in the nucleolar-like structure.

### Antisense proteins from other HTLV-1-related viruses do not interact with NPM1/B23

**Figure 3:**
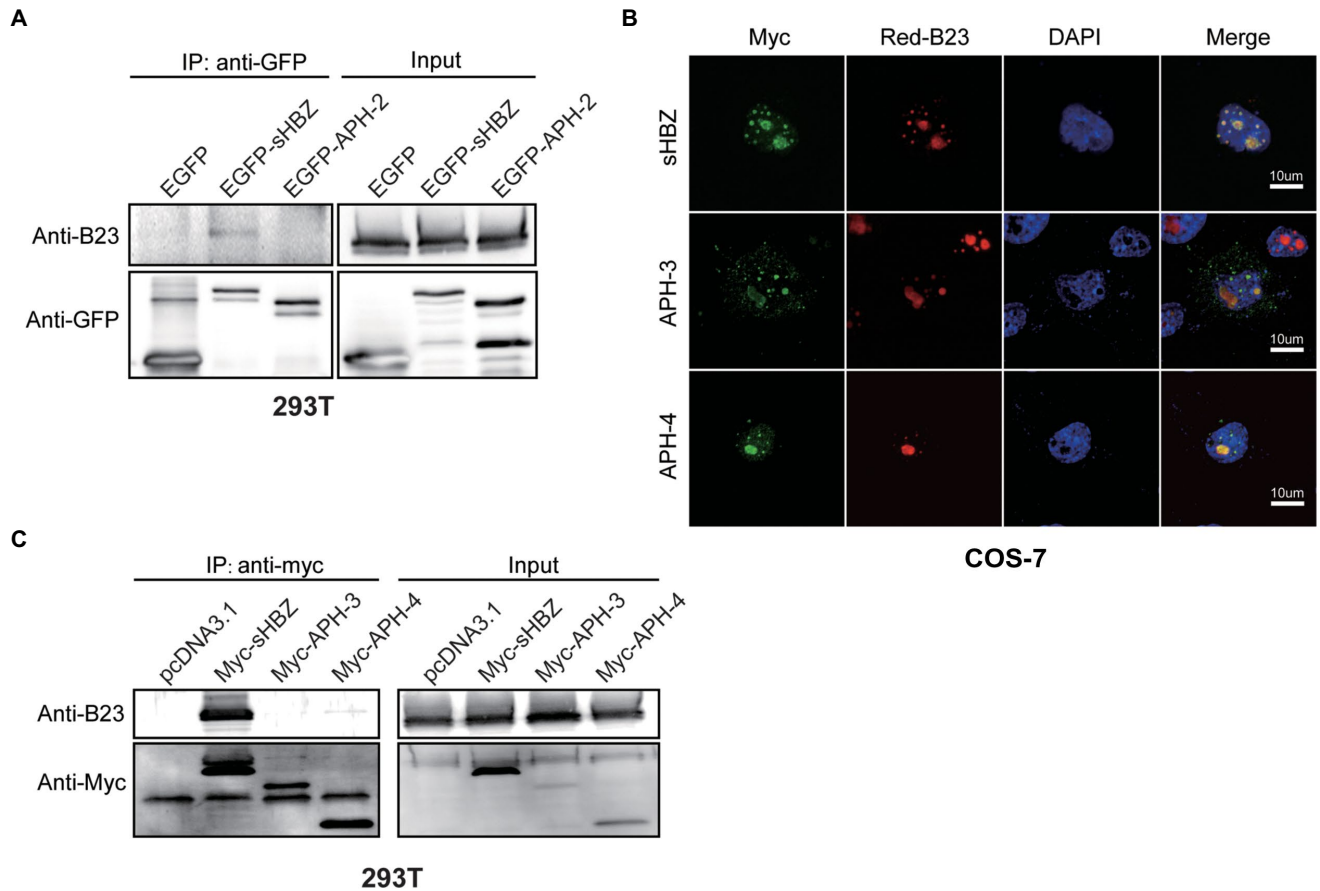
Comparison between sHBZ, APH-2, APH-3 and APH-4 for their association with NPM1/B23. 293 T cells were transfected with expression vectors for EGFP-fused sHBZ or APH-2 **(**vs. empty vector; **A)** or for Myc-tagged sHBZ, APH-3 and APH-4 **(**vs. empty vector; **C)**. Cell lysates were immunoprecipitated with anti-GFP or anti-Myc antibodies and analyzed by Western blot using anti-B23, anti-GFP or anti-Myc antibodies. **(B)** COS-7 cells were co-transfected with expression vectors for Myc-tagged sHBZ, APH-3 or APH-4 and pDsRed-B23. Cells were fixed and were analyzed by confocal microscopy with an anti-Myc antibody and stained with DAPI. The experiments were repeated three times.

We next sought to determine if NPM1/B23 could interact with antisense-transcript-encoded proteins of HTLV-2, HTLV-3 and HTLV-4 known as APH-2, APH-3 and APH-4. We have previously demonstrated that APH-2 is nuclear, but non-nucleolar (Halin et al., 2009). On the other hand, APH-3 and APH-4 both localizes to the nucleus and the nucleolus, although confocal microscopy analyses did reveal differences in localization in comparison to sHBZ (Larocque et al., 2011). 293 T cells were first transfected with expression vectors for GFP-sHBZ and GFP-APH-2 fusion proteins, followed by co-immunoprecipitation of cellular extracts with anti-GFP antibodies. Resulting Western blot analyses demonstrated that, unlike sHBZ, APH-2 did not co-immunoprecipitate with NPM1/B23 (Figure 3A).

We next tested if APH-3 and APH-4 could indeed associate with NPM1/B23. We conducted confocal microscopy analyses of COS-7 cells co-transfected with DsRed-B23 and Myc-tagged APH-3 or APH-4 expression vectors. Results confirmed our previous findings that NPM1/B23 co-localized with APH-3 and APH-4 in nucleoli (Figure 3B). However, when 293 T cells were transfected with expression vectors for Myc-tagged sHBZ, APH-3 and APH-4 and resulting extracts were co-immunoprecipitated with anti-Myc antibodies, no association between NPM1/B23 and APH-3 or APH-4 were convincingly demonstrated (Figure 3C). However, as previously reported, in these analyses, APH-4 and most markedly APH-3 were less abundant in transfected cells and the interaction between both proteins and NPM1/B23 might have not been detected in these experiments.

These results hence demonstrated that APH-2 (and APH-3 and APH-4) are not as potent to associate with NPM1/B23, even if strong co-localization with NPM1/B23 is apparent for APH-3 and APH-4, thereby suggesting that a specific sHBZ region is required for proper association.

### sHBZ associates with C23 without disturbing its interaction with NPM1/B23

**Figure 4:**
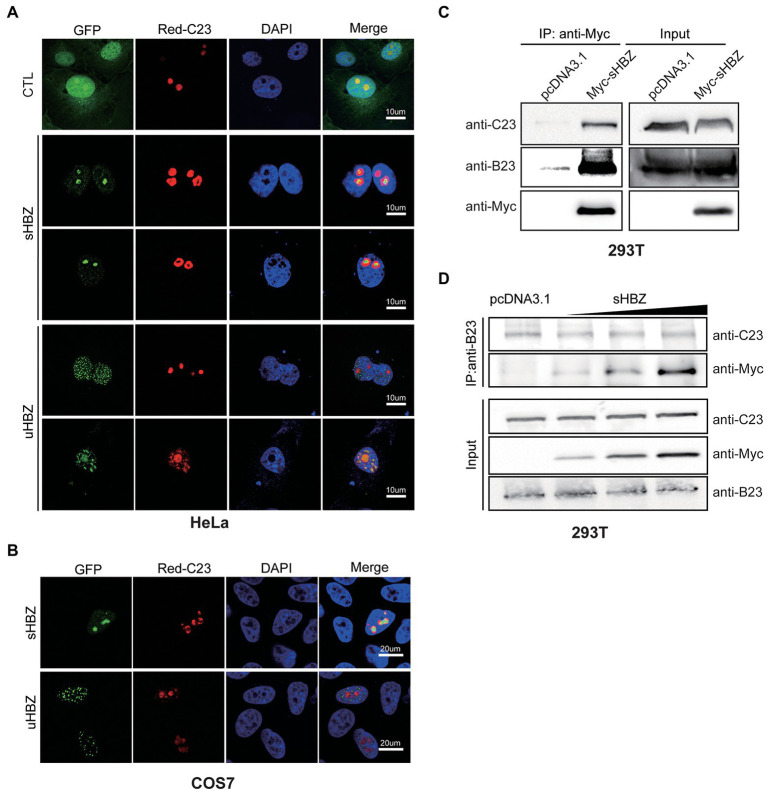
sHBZ is associated with C23 but does not impact its interaction with NPM1/B23. **(A–B)** HeLa **(A)** and COS-7 cells **(B)** were co-transfected with expression vectors for GFP-fused sHBZ or uHBZ (vs. pEGFP-C1; CTL) and pDsRed-C23. Fixed cells were analyzed by confocal microscopy following staining with DAPI. **(C)** 293 T cells were transfected with expression vectors for Myc-tagged sHBZ (vs. empty vector pcDNA3.1). Cell extracts were immunoprecipitated with anti-Myc antibodies. Immunoprecipitates and cell extracts were analyzed by Western blot using anti-C23, anti-B23 or anti-Myc antibodies. **(D)** 293 T cells were co-transfected with increasing concentrations of the Myc-tagged sHBZ expression vector. Cells were lysed and lysates were used for immunoprecipitation with anti-B23 antibodies. Immunoprecipitates and cell extracts were analysed by Western blot using anti-C23, anti-Myc or anti-B23 antibodies. The experiments were repeated three times.

We next investigated if the nucleolar C23 protein might also be part of the sHBZ-NPM1/B23 complex. Firstly, the localization of sHBZ and C23 were examined in COS-7 and HeLa cells co-transfected with Red-C23 and GFP-fused sHBZ or uHBZ expression vectors (Figures 4A,B). Confocal microscopy analyses indicated co-localization between C23 and both uHBZ and sHBZ in COS-7 and HeLa cells, although a more dominant co-localization was apparent for NPM1/B23. Strikingly, regions of HBZ-related fluorescence included areas in which C23 was not observed, especially in the nucleolar region. As co-localization was detected between sHBZ and C23, co-immunoprecipitation experiments were next conducted in transfected 293 T cells (Figure 4C). When extracts from 293 T cells expressing Myc-tagged sHBZ were immunoprecipitated with an anti-Myc antibody, signals for both C23 and B23 were detected along with sHBZ (Figure 4C). Similar results were obtained in sHBZ-expressing HeLa cells showing C23 association to the sHBZ-B23 complex (data not shown).

As previous studies have demonstrated that NPM1/B23 interacts with C23 and shuttles C23 to the nucleolus (Li et al., 1996; Liu and Yung, 1999), we were interested in evaluating if sHBZ could impact the interaction between NPM1/B23 and C23. 293 T cells were transfected with increasing amounts of the sHBZ expression vector and extracts were subjected to immunoprecipitation using an anti-B23 antibody. As shown in Figure 4D, levels of C23 co-immunoprecipitated with B23 remained steady regardless of the abundance of sHBZ.

These results hence suggested that C23 could associate with the sHBZ-B23 complex but was not outcompeted from its binding to NPM1/B23 by sHBZ.

### The HBZ basic regions are necessary for the association between sHBZ and NPM1/B23

**Figure 5:**
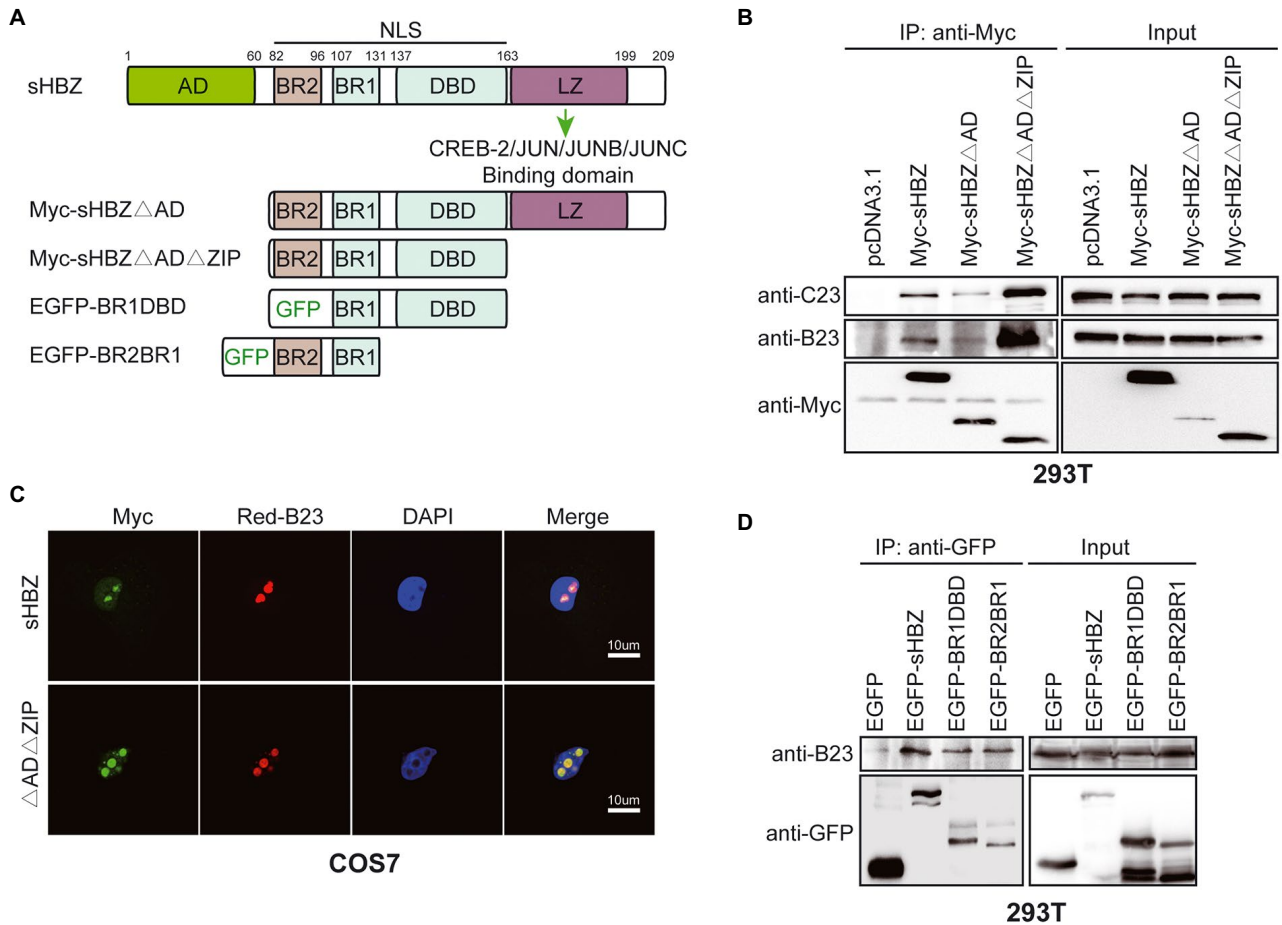
The basic regions of sHBZ are sufficient for its association with NPM1/B23**. (A)** Important domains/regions of sHBZ. **(B)** 293 T cells were transfected with Myc-tagged sHBZ, sHBZ∆AD, or sHBZ∆AD∆ZIP expression vectors. Cellular extracts were immunoprecipitated with anti-Myc antibodies. Immunoprecipitates or total cellular extracts were subsequently analysed by Western blot using anti-Myc, anti-C23 or anti-B23 antibodies. **(C)** COS-7 cells were co-transfected with sHBZ or sHBZ∆AD∆ZIP expression vectors and pDsRed-B23. Cells were fixed, stained with DAPI and analyzed by confocal microscopy. **(D)** 293 T cells were transfected with expression vectors for EGFP-fused sHBZ, sHBZ-BR1DBD or sHBZ-BR2BR1 (vs. pEGFP-C1). Cellular extracts were immunoprecipitated with anti-GFP antibodies. Immunoprecipitates or total cellular extracts were subsequently analyzed by Western blot using anti-GFP or anti-B23 antibodies. The experiments were repeated three times.

HBZ is comprised of several functional domains: the activation domain (AD), nuclear localisation signals (including basic region BR2, BR1 and DBD), and the Leucine Zipper region (Figure 5A; Barbeau et al., 2013). The LZ region has been shown to be a crucial region for protein–protein interaction, although other domains have also been implicated in many protein interactions (Clerc et al., 2008; Reinke et al., 2010; Zhao and Matsuoka, 2012). To determine which domain of HBZ is essential for its binding to NPM1/B23, expression vectors for Myc-tagged sHBZ, sHBZ∆AD and sHBZ∆AD∆ZIP were transfected in 293 T cells. Following immunoprecipitation with anti-Myc antibodies, Western blot analyses were performed. Both mutants and WT HBZ were able to associate with NPM1/B23 and C23, despite presenting different intensities. However, the sHBZ∆AD∆ZIP mutant seemed to harbor enhanced affinity toward both NPM1/B23 and C23 (Figure 5B). Interestingly, in transfected COS-7 cells, this mutant exhibited a more important nucleolar localization than for WT sHBZ with a concomitant higher co-localization with NPM1/B23 (Figure 5C). To attempt to refine the region required for NPM1/B23 interaction, we tested two previously described GFP-fused HBZ mutants containing either BR2-BR1 or BR1-DBD (Figure 5D; Hivin et al., 2005). Both mutants showed weaker but specific binding to NPM1/B23 upon analyses of anti-GFP-immunoprecipitated extracts from transfected 293 T cells and suggested that the shared BR1 region might be important for the association of sHBZ with NPM1/B23.

These data thus indicated that the basic regions of sHBZ are required for its association to NPM1/B23 and that the removal of the AD domain and ZIP region concomitantly induces higher NPM1/B23 association and nucleolar localization.

### The nucleic acid-binding domain of NPM1/B23 is important for its association with sHBZ

**Figure 6:**
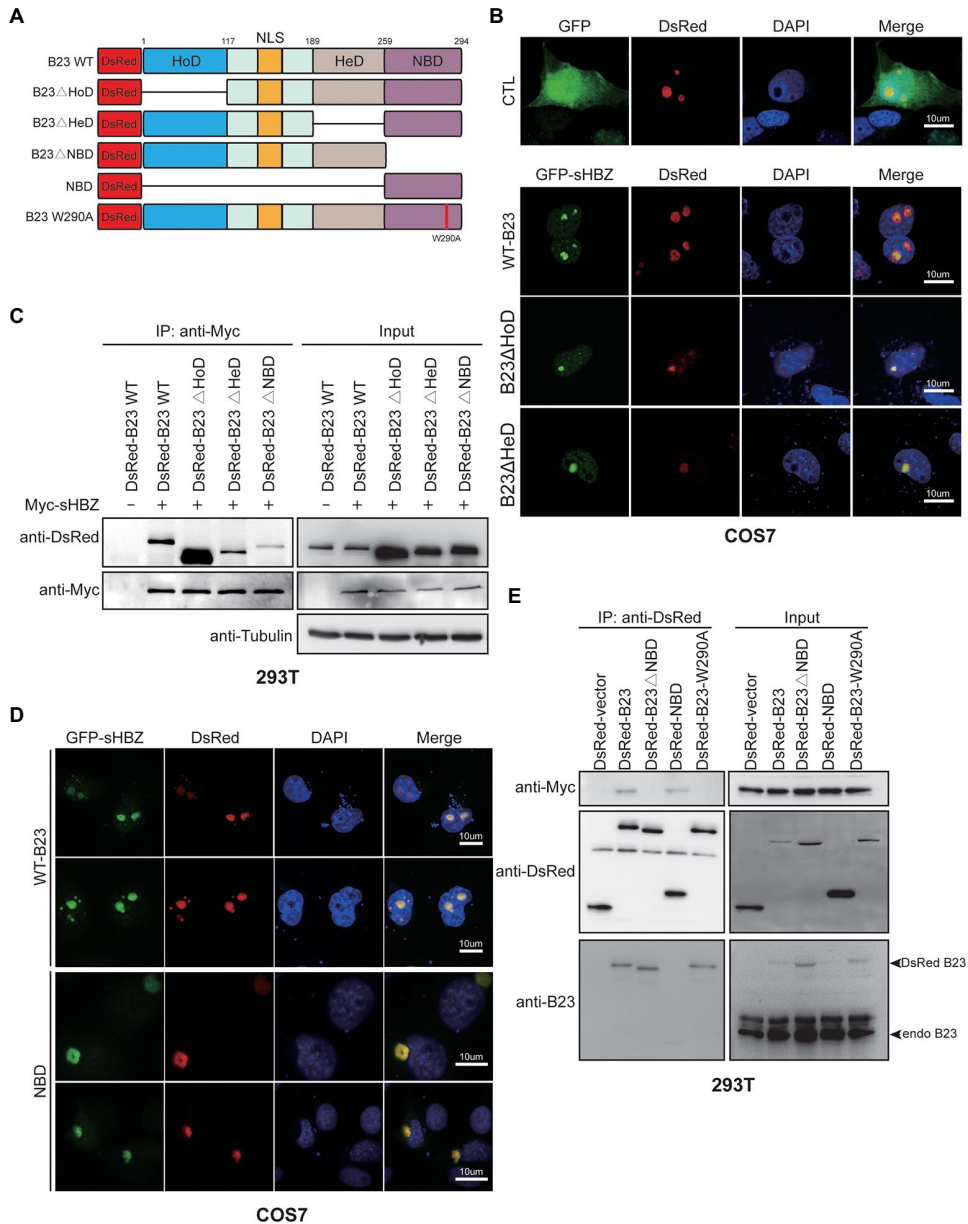
The RNA-binding domain of NPM1/B23 is required for its association with sHBZ**. (A)** Domains of NPM1/B23 and deletion/point mutants. **(B, D)** COS-7 cells were co-transfected with expression vectors for GFP-fused sHBZ and DsRed-fused B23 WT, B23∆HoD, B23∆HeD **(B)** or NBD **(D**; vs. empty vector pEGFP-C1: CTL**)**. Cells were fixed, stained with DAPI and analyzed by confocal microscopy. **(C–E)** 293 T cells were co-transfected with expression vectors for Myc-tagged sHBZ and wild-type or deletion/point mutants of NPM1/B23 fused to DsRed. Cellular extracts were immunoprecipitated with anti-Myc **(C)** or anti-DsRed antibodies **(E)**. Immunoprecipitates and total extracts were analyzed by Western blot with anti-Myc, anti-DsRed, anti-B23 or anti-tubulin antibodies. The experiments were repeated three times.

NPM1/B23 is characterized by the presence of a homo-oligomerization (HoD), an heterodimerization (HeD) and a nucleic acid-binding domain (NBD; Figure 6A; Okuwaki, 2008). In order to characterize which domain of NPM1/B23 is important for its association with sHBZ, deletion mutants were generated and co-localization with sHBZ was assessed by confocal microscopy in transfected COS-7 cells. As shown in Figure 6B, sHBZ co-localized to nucleoli with HoD-and HeD-deleted NPM1/B23 mutants. As expected, these deletion mutants co-immunoprecipitated with sHBZ in transfected 293 T cells (Figure 6C). However, in cells expressing the NBD deletion mutant, the association with sHBZ was strongly reduced, as assessed by co-immunoprecipitation. As expected, no sHBZ and NPM1/B23-specific signals were detected in anti-Myc immunoprecipitates when sHBZ was not expressed.

To further determine the role of NBD in the sHBZ/NPM1/B23 interaction, NBD fused to DsRed was expressed in COS-7 cells and, upon analysis by confocal microscopy, shown to strongly co-localize with sHBZ (Figure 6D). In transfected 293 T cells, as opposed to the NBD-deleted form of NPM1/B23, the NBD only-containing mutant was shown to be associated with sHBZ (Figure 6E). To validate that nucleolar localization is important for the association, a DsRed-B23 fusion protein with a point mutation at position 290 (B23 W290A), known to be non-nucleolar (Falini et al., 2006; Grummitt et al., 2008), was co-expressed with sHBZ in 293 T cells and analysed by co-immunoprecipitation. As depicted in Figure 6E, association of this mutant with sHBZ was not observed, despite its successful immunoprecipitation.

These data hence provided strong evidence that the NBD domain is responsible for the association of NPM1/B23 to sHBZ and further confirmed the importance of nucleolar localization for the interaction.

### sHBZ associates with NPM1/B23 in an RNA-sensitive manner and binds to its own mRNA

**Figure 7:**
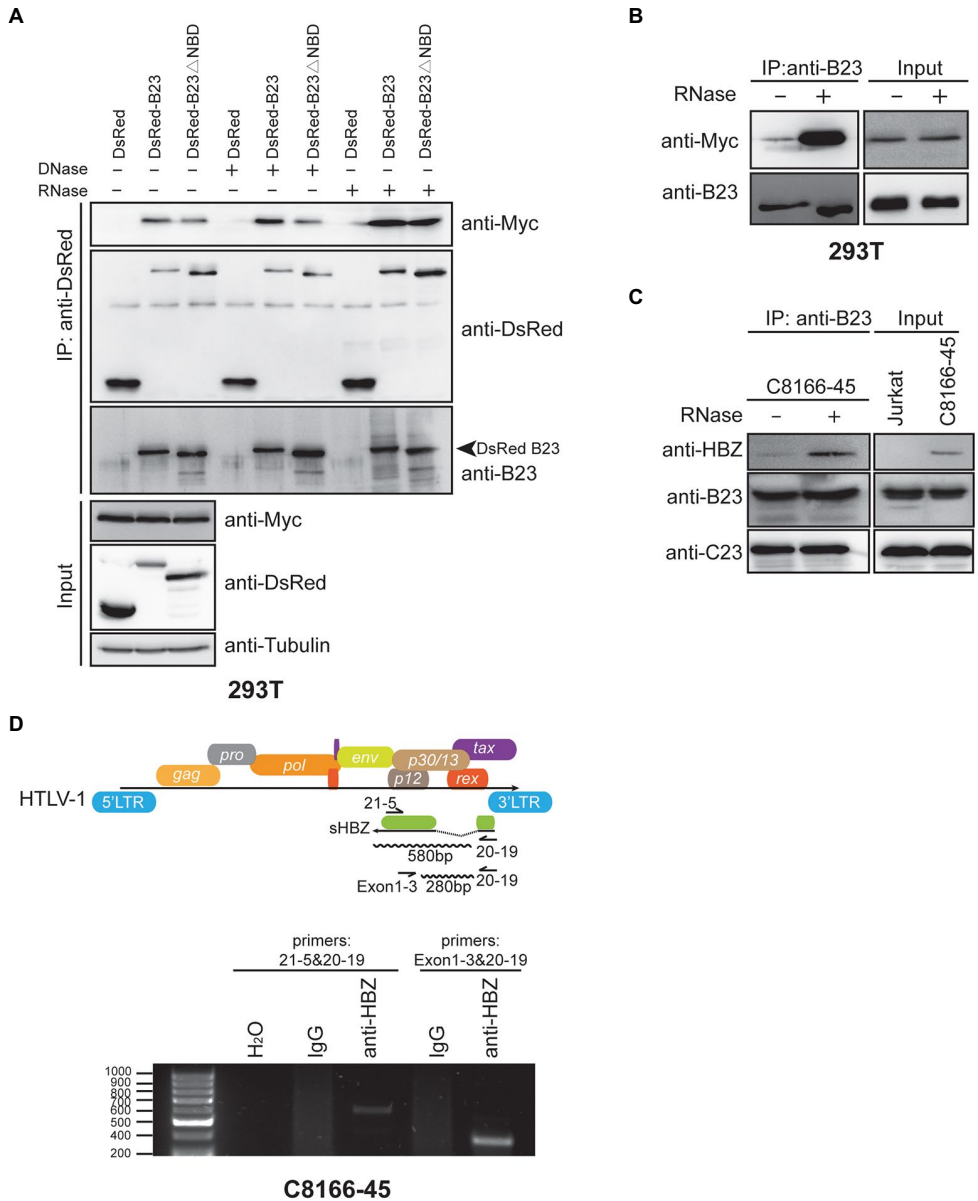
Association of RNA to the HBZ-NPM1/B23 complex hampers their association. **(A–B)** 293 T cells were transfected with the expression vector for Myc-tagged sHBZ alone **(B)** or in the presence of pDsRed-B23, pDsRed-B23∆NBD or pDsRed **(A)**. Cell extracts were immunoprecipitated with anti-DsRed **(A)** or anti-B23 **(B)** antibodies, and next treated with DNaseI or RNaseA before elution. Immunoprecipitated and total extracts were analysed by Western blot using anti-Myc, anti-DsRed, anti-B23 or anti-tubulin antibodies. **(C)** Extracts from Jurkat and C8166-45 cells were immunoprecipitated with anti-B23 antibodies and treated or not with RNaseA before elution. Immunoprecipitated and total extracts were analysed by Western blot using anti-HBZ, anti-B23 and anti-C23 antibodies. **(D)** C8166-45 cell lysates were immunoprecipitated with either control IgG or anti-HBZ antibodies. Isolated RNA from immunoprecipitated samples were analyzed for *shbz* transcripts by RT-PCR with indicated primers in the top of the panel (size of expected amplicons are also indicated; negative control H_2_O). The experiments were repeated twice.

As the above results demonstrated that the NPM1/B23 NBD domain was sufficient for its association with sHBZ, we were next interested in determining if DNA or RNA could be involved in the formation of the sHBZ-NPM1/B23 complex. To address this possibility, extracts from 293 T cells co-transfected with expression vectors for sHBZ and DsRed-B23 or DsRed-B23∆NBD were co-immunoprecipitated with anti-DsRed followed by DNase or RNase treatment before elution of the immunoprecipitated complex (Figure 7A). As demonstrated above, the interaction of sHBZ was reduced with the NBD-deleted NPM1/B23 mutant compared to WT NPM1/B23. After DNase treatment, no significant change in the intensity of the interaction between sHBZ and NPM1/B23 was apparent, as revealed by the analysis of the immunoprecipitated samples. Surprisingly, when immunoprecipitated extracts were treated with RNase, NPM1/B23 was more strongly associated to sHBZ than without treatment. Unexpectedly, the interaction was also increased with the NBD-deleted NPM1/B23 mutant (see “Discussion”). A similar important increase in the association between these proteins in the presence of RNase was revealed in transfected 293 T in the absence of overexpressed DsRed-B23 (Figure 7B). We further extended these analyses to C8166-45 cells. Extracts were thus co-immunoprecipitated with anti-B23 antibodies and treated or not with RNase (Figure 7C). Again, RNase treatment led to stronger association between sHBZ and NPM1/B23. Interestingly, when C23 was analysed in the complex, comparable levels were detected regardless of the addition of RNase treatment.

Since NPM1/B23 is known to interact with RNA and that RNAse treatment increases the association between sHBZ and NPM1/B23, we thereby suspected that sHBZ might be part of a complex in which RNA disfavors specific interaction and that sHBZ might in fact be a potential RNA-binding protein, especially knowing that its DBD region has not been shown to bind to DNA. To test this, we focused our attention on *shbz* transcripts, as previous studies had suggested that this viral transcript was more prone to nuclear retention in HTLV-1-infected cells (Rende et al., 2011; Ma et al., 2021). RNA immunoprecipitation (RIP) was performed with anti-HBZ antibodies on C8166-45 cell extracts. Isolated RNA was next analysed by RT-PCR using primers specific for the detection of spliced transcripts (shown in Figure 7D). As depicted in Figure 7D, *shbz* mRNA was specifically detected in anti-HBZ immunoprecipitates for each tested primer pair, while no equivalent signals were observed in control immunoprecipitation (IgG). Sequencing confirmed that these signals represented spliced *hbz* mRNA. Anti-HBZ immunoprecipitated RNA were also negative when analysed for the presence of *GAPDH* mRNA by RT-PCR.

These results hence suggested that the association between sHBZ and NPM1/B23 was competed by RNA and that, like NPM1/B23, sHBZ could associate with RNA, which included its own spliced transcript.

### *NPM1/B23* expression alters sHBZ expression with a potential cooperation toward cell transformation

**Figure 8:**
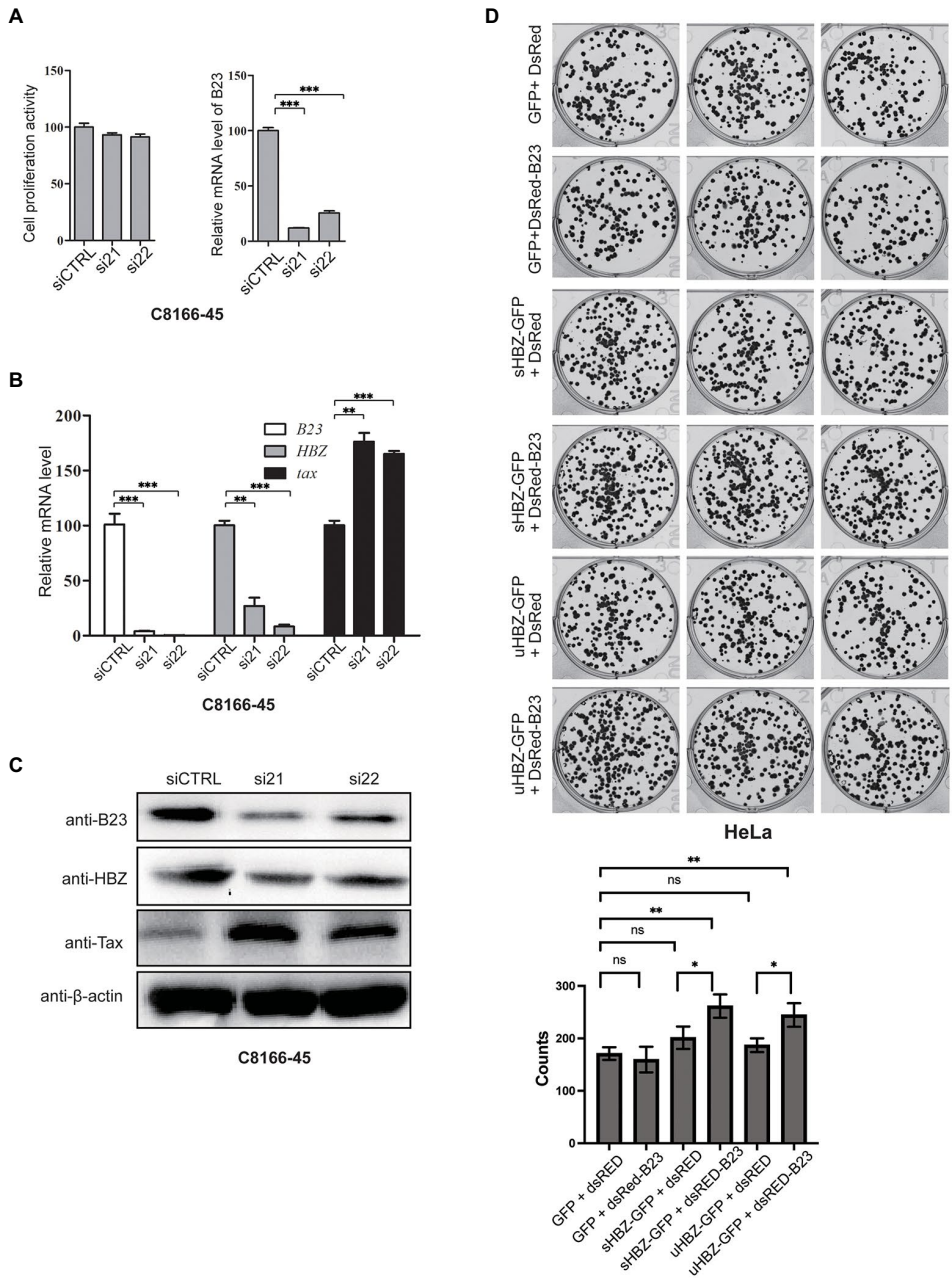
*NPM1*/B23 expression modulates viral gene expression and alters cell transformation. **(A)** C8166-45 cells were transfected with *NPM1*/B23-specific siRNA (si21 and si22) vs. control siRNA (siCTRL). After 72 h, cell viability was determined by Cell Counting Kit-8 and *NPM1*/B23 expression levels were quantified by qRT-PCR. **(B)** C8166-45 cells transfected with *NPM1*/B23 siRNAs or scrambled siRNA were analyzed for *NPM1*/B23, *hbz* and *tax* expression by qRT-PCR at 72 h post-transfection. The data are presented as mean ± SEM (*n* = 3). Values for siCTRL-transfected cells were set as 100%. **(C)** NPM1/B23, Tax and HBZ protein levels were analyzed by Western blot 72 h after siRNA transfection. β-actin was analyzed as a loading control. **(D)** HeLa cells were transfected with sHBZ, uHBZ and/or DsRed-B23 expressing vectors (vs. corresponding empty vectors). After 24 h, cells were counted, and 100 cells were seeded into each well of 6 well-plates for each group in soft agar solution. At 15-day post-transfection, plates were read and analyzed with a GloMax plate reader. Experiments were performed in triplicates and repeated twice. The data are presented as mean ± SEM (*n* = 3), ns = non-significant, *p < 0.05 **p ≤ 0.01 and ***p ≤ 0.001 (ANOVA comparisons tests).

To investigate the potential function of the sHBZ-NPM1/B23 association in HTLV-1-infected cells, we next sought to repress NPM1/B23 expression. We first attempted to knock-out the *NPM1* gene in C8166-45 by CRISPR/Cas9: no clones with lack of expression were recuperated, likely due to the essential role of this protein for cell survival. We thus relied on a silencing approach of *NPM1*/B23 expression in chronically infected cell lines using two different siRNA (Figure 8A). Our data showed that partial downregulation of *NPM1* was observed in C8166-45 cells, although little effect was noted on cell viability. Interestingly, *NPM1*/B23 silencing led to a reduction in sHBZ expression (mRNA and protein levels) with a concomitant increase in Tax expression (Figures 8B,C). To determine if NPM1/B23-sHBZ association could promote cell transformation, a soft agar growth assay was performed in HeLa cells, which were transfected with expression vectors for GFP-sHBZ, GFP-uHBZ, DsRed-B23 and/or empty vectors. Resulting clone counts showed that concomitant expression of either uHBZ or sHBZ with NPM1/B23 led to an increase in cell transformation (Figure 8D), although sHBZ showed a slightly higher number of clones (non-significant). Of note, NPM1/B23 alone did not lead to significant changes in clone numbers.

These results thus suggest that nucleolar sHBZ through its interaction with NPM1/B23 could act upon HBZ expression and subsequently Tax production with a potential link with cell transformation.

## Discussion

Several DNA and RNA viruses are known to make use of the nucleolus for efficient replication. Several viral proteins in fact transit to the nucleolus, while for certain viruses, infection can lead to change in the localisation of nucleolar components, or reversibly import cytoplasmic constituents into the nucleus or nucleolus in order to enhance viral fitness (Hiscox, 2002; Greco, 2009). NPM1/B23 has been previously reported to be a mediator of these associations between the nucleolus and viruses in certain cases, lending support to replication and acting on functional properties of proteins, such as HIV-1 Rev and Tat, and HTLV-1 Rex (Fankhauser et al., 1991; Adachi et al., 1993; Li, 1997). Herein, we have focused on HBZ, an HTLV-1 protein previously shown to localize to the nucleolus. Our findings demonstrate that HBZ associates with NPM1/B23, that RNA hampers complex formation, and that this association could play a role in transforming properties of sHBZ.

Based on the nucleolar localization of sHBZ and the previously reported implication of NPM1/B23 in various leukemic cancers (Pandolfi, 1996; Yoneda-Kato et al., 1996; Grisendi et al., 2006; Kunchala et al., 2017), we focused our attention on its potential interaction with both HBZ isoforms. We first determined if NPM1/B23 expression was impacted in PBMCs from ATL and HAM/TSP patients when compared to asymptomatic HTLV-1-infected individuals. A significant increase in *NPM1*/B23 expression was observed in PBMCs from ATL patients. Given that expression of *NPM1*/B23 was more important in PBMCs from ATL patients, we thus speculated that a functional association could be at play between both nuclear proteins. Hence, this led us to more precisely assess the potential association between HBZ and NPM1/B23. Confocal experiments and co-immunoprecipitation experiments indeed revealed association of NPM1/B23 with both HBZ isoforms. Since uHBZ does not localize to the nucleolus, these results suggested that NPM1/B23 interaction also occurs externally to the nucleolus, which is reminiscent of the C23-NPM1/B23 interaction also occurring outside of the nucleolus (Liu and Yung, 1999). However, of note, when NPM1/B23 was over-expressed, sHBZ seemed to localize to nucleoli to a greater extent. This was more clearly observed in COS-7 cells overexpressing NPM1/B23 and uHBZ, the latter being prominently co-localized with NPM1/B23 to the nucleolus. These results suggest that NPM1/B23, being a shuttling protein, may help sHBZ to be retained in the nucleoli in a similar manner to other cellular and viral proteins, such as HIV-1 Tat (Li, 1997; Greco, 2009).

Our analyses have further validated the association of sHBZ with NPM1/B23 in transfected T cell lines, chronically infected T cell lines and freshly infected HeLa cells by confocal microscopy and co-immunoprecipitation experiments. Although the exact isoform detected in infected cells was not determined in these analyses, previous studies have indicated that the sHBZ isoform was the main isoform detected in ATL cell lines (Murata et al., 2006), which is further supported by the nucleolar-like distribution of most of our HBZ signals. However, recent reports have argued that, in ATL cells (and thereby in more relevant HBZ-expressing cell context), HBZ did not localize to the nucleolus, as determined by confocal microscopy analyses and C23 labeling (Raval et al., 2015). Moreover, a more recent study has suggested that HBZ only localized in the cytoplasm of infected cells from asymptomatic carriers and HAM/TSP patients (Forlani et al., 2019). Although it is possible that the HBZ-NPM1/B23 interaction in the context of infected cells might occur in non-nucleolar compartments, our results nonetheless argue that HBZ in the context of infected cells localizes to the nucleus and the nucleolus, which agrees with previous studies. Importantly, a previous report has revealed that ATL cells can be devoid of nucleoli (Kamihira et al., 1993), and might not easily lead to a positive nucleolar distribution for HBZ, as those observed in non-ATL cells. Regardless of these contradictory findings, our results confirmed the association of HBZ with NPM1/B23 in relevant expression levels.

An important finding in this study was that APH-2 and possibly APH-3 and APH-4 were unable to co-immunoprecipitate NPM1/B23, despite the fact that APH-4 is a nucleolar protein. We and others have previously reported on functional differences between APH proteins and sHBZ in addition to differences in subcellular localization (Halin et al., 2009; Larocque et al., 2011; Marban et al., 2012; Yin et al., 2012; Larocque et al., 2014; Panfil et al., 2016). These functional differences might be dependent on the cellular localization of these proteins and might also be attributed to their varying affinities toward interacting partners. However, whether the lack of interaction of NPM1/B23 with antisense proteins of other HTLV viruses is responsible for some of the divergent effects of these various antisense proteins over the activation potential of different transcription factors remains to be determined. Further experiments are underway to clarify these results, especially those related to APH-3 and APH-4, which have strong intrinsic instability and therefore lead to less firm conclusions.

An important interacting partner of NPM1/B23 is C23. Among its many functions, C23 plays a crucial role in cell proliferation and survival (Ugrinova et al., 2007). In fact, previous studies have used C23 to demonstrate the nucleolar localization of sHBZ and deleted mutants of uHBZ (Hivin et al., 2005; Murata et al., 2006; Hivin et al., 2007). Based on our results showing that C23 only partially co-localized with sHBZ, we suggest that C23 is part of the sHBZ-associated complex due to its NPM1/B23-interacting properties, which would explain why the NPM1/B23-C23 interaction is not outcompeted by sHBZ. Furthermore, previous studies indicated that C23 and NPM1/B23 do not completely localize to the same compartment of the nucleolus (Biggiogera et al., 1989). Our results showed that the region important for NPM1/B23 interaction with sHBZ was different from the domain implicated in its association with C23 (Li et al., 1996). Indeed, analyses of deletion mutants of NPM1/B23 allowed us to conclude that the NBD domain was important for its association with sHBZ. Of note, the residual interaction observed between the NBD-deleted mutant with sHBZ in some co-immunoprecipitation experiments might be due to its capacity to interact with endogenous NPM1/B23 through its HoD domain. This could partially redirect this mutant to the nucleolus and promote association with sHBZ in multimers composed of endogenous NPM1/B23 and the NPM1/B23 mutant. In fact, results from immunoprecipitation experiments indeed argued for interaction between NBD-deleted B23 mutant and endogenous B23 (data not shown).

We have further shown that the association of sHBZ with NPM1/B23 likely depends on the BR1 and/or BR2 basic regions. Interestingly, these regions are also essential for nuclear and nucleolar localization and are likely to independently impact the extent of sHBZ-NPM1/B23 association. Deletion of AD and bZIP domains of sHBZ led to stronger interaction with NPM1/B23 with more intense nucleolar localization, as previously demonstrated (Hivin et al., 2005). These results suggest that interaction with other cellular proteins through these domains may compete for the nucleolar localization of sHBZ and/or NPM1/B23 interaction. As the two known HBZ isoforms only differ in their first amino acids (four vs. seven for sHBZ and uHBZ, respectively), it is expected that both have the potential to interact with NPM1/B23, as we have shown in this study. More analyses are needed to precisely decipher the regions and amino acids required from both isoforms for their interaction and comparison of BR1 and BR2 sequences between sHBZ and antisense proteins from other HTLV viruses will be helpful.

**Figure 9:**
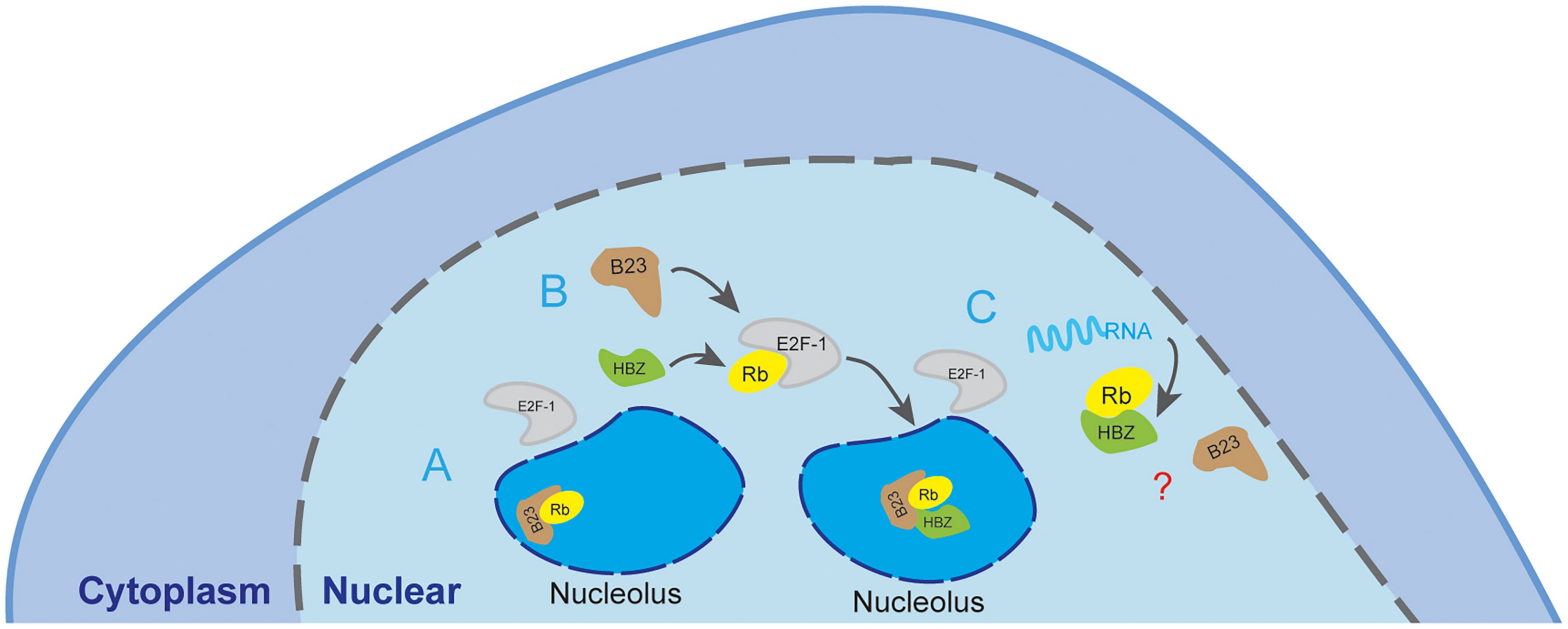
Proposed model of the association between NPM1/B23 and sHBZ. Based on our results, sHBZ would interact with NPM1/B23 in the nucleolar region of HTLV-1-infected cells. **(A)** In a non-infected cell, the activation of the E2F-1 transcription factor involves phosphorylation of Rb and its sequestration in the nucleolus. NPM1/B23 helps in this sequestration by direct interaction with pRb. **(B)** In HTLV-1 infected cells, we speculate that sHBZ forms a ternary complex with NPM1/B23 and Rb (potentially phosphorylated) and leads to sequestration of Rb in the nucleolus through an alternative B23-based mechanism. **(C)** As our results further lead to the speculation that this ternary complex could be outcompeted by bound RNA, we propose that binding of *shbz* transcripts to HBZ could interfere with the formation of the HBZ-NPM1/B23 complex, subsequently leading to modulation of E2F-1 activation *via* alteration of Rb, although the outcome remains unknown.

We have further shown that the interaction between the NBD domain of NPM1/B23 and sHBZ was potently competed by RNA. As RNase treatment was performed in lysed extracts, concomitant binding of RNA likely destabilizes NPM1/B23 interaction with sHBZ. The additional discovered interaction between HBZ and its transcript is of important interest. It is hence possible that, through this association, HBZ could auto regulate its own expression by modulating nuclear export of its transcript. Furthermore, the interaction between sHBZ and its own mRNA could be linked to ATL development. Indeed, *hbz* mRNA was reported to induce IL-2-independent T-cell growth, but also to enhance transcription of E2F-1 and downstream targets, which has also been shown to be regulated by NPM1/B23 (Lin et al., 2006; Satou et al., 2006; Matsuoka and Green, 2009; Mitobe et al., 2015). Hence, as it has been proposed that NPM1/B23 association to Rb sequesters it in the nucleolus thereby permitting E2F to be activated, it can be speculated that HBZ interaction to NPM1/B23 alters its association to Rb. If *hbz* RNA interferes with HBZ-NPM1/B23 interaction, then it would modulate the impact of HBZ on E2F-1 activation (Figure 9). In this sense, it is further interesting to note that we have detected higher levels of NPM1/B23 transcripts in PBMC from ATL patients vs. other HTLV-1-infected individuals.

We have further examined this new association in terms of its potential impact on properties attributed to sHBZ. Our results demonstrated that silencing of NPM1/B23 expression in the chronically infected C8166-45 cell line resulted in downregulation of HBZ expression with concomitant increase in Tax level. Of note, no differences in cell viability of *NPM1/B23*-silenced C8166-45 cells (and MT-2 cells, unpublished result) vs. control siRNA were observed. Although unexpected, this might be caused by the concomitant increase in Tax expression. These results are not directly assessing the HBZ-NPM1/B23 complex, but they do suggest that NPM1/B23 could act upon the stability of sHBZ and limit its delocalization to nuclear speckles, thereby acting on the positive auto regulatory loop toward its expression and the negative regulation of Tax expression (Gaudray et al., 2002; Gazon et al., 2012). On the other hand, as reduced *shbz* RNA is also indicated, NPM1/B23 might instead be acting directly on the transcript.

Given that knock-out of *NPM1*/B23 gene was not possible, we instead investigated the potential implication of NPM1/B23 on sHBZ-mediated transformation in HeLa cells through transfection of expression vectors. Our results argue that when NPM1/B23 is co-expressed with sHBZ, anchorage-independent growth shows the highest value, while results showed that uHBZ also led to higher level in the presence of NPM1/B23. This might be in line with our observation that NPM1/B23 overexpression led to higher nucleolar distribution of uHBZ. More experiments are needed to better understand the importance of the sHBZ-NPM1/B23 complex and the transforming features of sHBZ in relation to HBZ stability. Furthermore, we need to appropriately distinguish between a direct effect of the complex on cell transformation versus a potential indirect effect *via* sHBZ-NPM1/B23-dependent higher sHBZ expression which could also impact this process. The involvement of RNA in the regulation of this complex will also require further experiments.

In conclusion, for the first time, our study demonstrates that NPM1/B23 is a potential interacting partner of HBZ. We speculate that a nucleolar distribution and a strong interaction with NPM1/B23 might be an important feature of sHBZ to induce IL-2-independent T-cell proliferation, an attribute, which is not shared with non-proliferation-inducing and non-nucleolar uHBZ and APH-2. Alternatively, other functional properties of sHBZ might also be attributed or modulated by the capacity of this viral protein to interact with NPM1/B23 in the nucleolus. Gaining a better understanding of how sHBZ interacts with NPM1/B23 and the outcome of this interaction at the cellular level will provide a better understanding of the link between sHBZ and ATL development.

## Materials and methods

### Cell lines

293 T, COS-7 and HeLa cells were cultured in DMEM supplemented with 10% fetal bovine serum (FBS) (PAA Laboratories Inc., Toronto, Canada). Jurkat, SupT1, H9, MT2 and C8166-45 cells were maintained in supplemented RPMI-1640 medium. All cell lines were incubated in 5% CO_2_ at 37°C.

### HTLV-1 infection of HeLa cells

Infection of HeLa cells was performed, as previously described (Liu et al., 2008; Philip et al., 2014). HeLa cells were first seeded at 5 × 10^5^/ml, incubated overnight and then co-cultured with Jurkat or MT2 cells (1.5 × 10^6^/ml) for 16 h. MT2 cells were subsequently removed by several washes with supplemented RPMI-1640. HeLa cells were then cultured for 5 days and used for further analyses.

### PBMC and CD4+ T cell isolation

PBMCs were isolated from blood samples of non-infected donors and HTLV-1-infected patients (asymptomatic (AC)), ATL, and HAM/TSP patients, including a Chinese patient (Gao et al., 2018) using the MACSprep™ PBMC Isolation Kit according to manufacturer’s instructions (Miltenyi Biotec Inc). CD4+ T cells were further isolated from PBMC samples from non-infected and the HAM/TSP Chinese patient with the CD4+ T Cell Isolation Kit (Miltenyi Biotec Inc) and used for confocal microscopy. All experiments and analyses using the patient samples were approved by the Medical Research Ethics Committee of the Second Affiliated Hospital of Fujian Medical University and Fujian Province Government (2016-SAH-FMU-No.48). Additional blood samples from HTLV-1-infected patients and non-infected donors were obtained from the CHU of Martinique. Isolated PBMC samples (11 from AC individual, 11 from HAM/TSP patients and 11 from ATL patients) were stored for research purpose at the Center of Biological Resources of Martinique (CeRBiM). HTLV-1 AC patients were recruited according to World Health Organization (WHO) criteria. AC had no neurologic or hematological symptoms. According to the French Bioethics laws, the collection of samples from AC and ATL has been approved by the ethic committee of the French Ministry of Research. Because the protocol is non-interventional, no informed consent was required, as stated by the French Public Health code and therefore the study was conducted anonymously.

### Plasmid constructs

Expression vectors for sHBZ and uHBZ (provided by Dr. J.M. Mesnard, Université de Montpellier 1, Montpellier, France), APH-2 (pGFP-APH-2; provided by Dr. R. Mahieux, ENS Lyon, France), APH-3 and APH-4 have been previously described and express GFP fusion or Myc-tagged proteins (pMyc/His-spHBZ, pMyc/His-uHBZ, pMycAPH-3, pMycAPH-4, pAPH-3-GFP and pAPH-4-GFP; Hivin et al., 2007; Halin et al., 2009; Larocque et al., 2011). The pMyc/His-HBZ∆AD and pMyc/His-HBZ∆AD∆ZIP vectors have also been previously reported and express different deletion mutants of HBZ (Hivin et al., 2007). The pDsRed-nucleolin and pDsRed-B23 vectors were generous gifts from Dr. D. Archambault (Université du Québec à Montréal, Montréal, Canada) and, respectively, express C23 and NPM1/B23 fused to DsRed (Passos-Castilho et al., 2018). Deletions and point mutations in the pDsRed-B23 vector were generated using the Phusion™ Site-Directed Mutagenesis Kit (Thermofisher Scientific Inc., Waltham MA) according to manufacturer’s instructions and the following primer pairs: 5′-GCTGTGGAG GAAGAT GCAGA-3′ and 5′-GTACCGTCG ACTGCAGAATTC-3′ for pDsRed-B23-∆HoD; 5′-TCTCTTCC CAAAGTGGAA GCC-3′ and 5′-AGCTTC CTCATCAT CAAAAT CATCA-3′ for pDsRed-B23-∆HeD; 5′-TAATC TAGAG GATCCAC CGGATCT-3′ and 5′-ACCAC CTTTTTC TATACTT GCTTGC-3′ for pDsRed-B23-∆NBD; 5′-TCTC TTCCCAAA GTGGAAGC CAAATTC-3′ and 5′-CATGGT ACCGTCG ACTGCAGA ATTCG-3′ for pDsRed-NBD (B23); 5′-GGCTAT TCAAGATCT CTGGCAG GCGAGGA AGTCTCTT TAA-3′ and 5′-TTAAAGA GACTTC CTCGCCTGC CAGAG ATCTTGAAT AGCC-3′ for pDsRed-B23-W290A.

### siRNA transfection

Control siRNA (#SIC001, Sigma) and siRNAs targeting NPM1/B23 (siRNA21 (SI049500010): CACCAGT GGTCTTAAG GTTGA and siRNA22 (SI04949994): AAGGACAAG AATCCT TCAAGA) were ordered from Qiagen and transfected into MT-2 and C8166-45 cells with Neon Transfection System (Invitrogen), according to manufacturer’s instructions. After 24 h, cells were harvested for further analyses.

### qRT-PCR analysis

Total RNA was isolated using the Trizol reagent (Invitrogen), as previously described (Terol et al., 2017). After reverse transcription (RT, High-Capacity cDNA Reverse Transcription, Thermofisher Scientific Inc.), transcript levels were assessed by qPCR analysis using the SYBR green PCR master mix (#04707516001, Roche Diagnostics) and gene-specific primer sets. PCR cycles were set as follow: 95°C, 5 min, followed by 40 cycles at 95°C for 5 s, 60°C for 10 s, 72°C for 30 s, 95°C for 5 s). Data were analyzed using the LightCycler®480 Software (Roche Diagnostics). The relative expression levels were calculated for each gene using the ΔΔCt method and normalized to the house-keeping gene HPRT-1. The following forward/reverse primers purchased from BioRad were used for these analyses: NPM1/B23 5′-GCTGGTGCAAAGGATGAGTT-3′ and 5′-AAGGGAAACCGTTGGCTGTA-3′; Tax 5′-CCGGCGCTGCTCTCATCCCGGT-3′ and 5′-GGCCGAACATAGTCCCCCAGAG-3′; HBZ: 5′-AGAACGCGACTCAACCGG-3′ and 5′-TGACACAGGCAAGCATCGA-3′; HPRT-1 (housekeeping gene): 5′-ATGGGAAGGTGAAGGTCGG-3′ and 5′-TGGAGGGATCTCGCTCCTGG-3′.

### Cell transfection

Cells (5 × 10^4^ COS-7 and 5 × 10^6^ 293 T) were plated for 24 h prior to transfection using the polyethyenimine (PEI) reagent with 0.25 μg and 6 μg of DNA, respectively at a 1:9 DNA:PEI ratio (Polysciences Inc). HeLa cells and SupT1 cells were transfected with Lipofectamine 2000 (Invitrogen) following the manufacturer’s protocol. After 6 to 12 h, cells were washed and replenished with fresh supplemented media. Cells were analysed from 24 to 48 h post-transfection.

### Immunoprecipitation experiments

Cellular extracts were prepared by resuspending cells in lysis buffer (50 mM Tris–HCl pH 8, 100 mM NaCl, 1 mM EDTA, 1% Triton). Immunoprecipitation was performed using Dynabeads Protein G (Invitrogen) according to manufacturer’s instructions. Briefly, 40 μl of Dynabeads Protein G were incubated 2 h at room temperature with 5 μg anti-Myc (#sc-40, Santa Cruz Biotechnology Inc., Santa Cruz CA), 4 μg anti-B23 (#sc-5,564, Santa Cruz Biotechnology Inc.), 4 μg anti-DsRed (Santa Cruz Biotechnology Inc.) or 4 μg anti-GFP antibodies. Cell extracts were then incubated overnight at 4°C with the antibody-bead complex. For certain experiments, RNaseA (100 μg/ml; #RB0473, Biobasic Inc., Markham, Canada) or DNaseI (10 μg/ml; #10104159001, Sigma-Aldrich Canada), was added to the antibody-bead complex and incubated for 20 min at room temperature. Bound fractions were eluted with 20 μl of loading buffer and then analysed by Western blot.

### Western blot

Immunoprecipitated or total extracts were separated on a 12% SDS-PAGE and transferred to a PVDF membrane (Millipore, Mississauga, Canada). Membranes were blocked in PBS/5% milk or PBS/0.3% BSA and incubated with anti-HBZ (1:2,000; Gaudray et al., 2002), anti-B23 (1:500, Sigma-Aldrich Canada Co., Oakville, Canada), anti-C23 (1:500, #sc-13,057, Santa Cruz Biotechnology Inc.), anti-DsRed (1:1000,#sc-101,526, Santa Cruz Biotechnology Inc), anti-GFP-HRP (1:1,000, #sc-9,996, Santa Cruz Biotechnology Inc.), anti-Tubulin (1:5,000, #T5168, Sigma-Aldrich Canada Co.), anti-β-Actin (1:5,000, #sc-47,778, Santa Cruz Biotechnology Inc.), anti-Tax (1:2,000, #sc-57,872, Santa Cruz Biotechnology Inc), or anti-Myc (1:250) antibodies overnight. After several washes, membranes were incubated with HRP-conjugated sheep anti-rabbit IgG antibodies (1:5,000, #5220–0336, Seracare, Milford MA) or anti-mouse IgG antibodies (1:5,000, #5220–0338, Seracare) for 2 h, washed several times and incubated with the BM Chemiluminescence Blotting Substrate. Membranes were analysed using the Fusion FX7 device.

### Confocal microscopy

Cells were washed three times with PBS and fixed with PFA 4% followed by permeabilization with 0.5% Triton. Infected HeLa, C8166-45 and CD4+ T cells were additionally treated with trypsin (2.5 μg/ml) for 5 min at 37°C prior analyses for endogenous NPM1/B23, as previously performed (Svistunova et al., 2012). Cells were then washed with cold PBS three times and incubated overnight in the presence of mouse anti-HBZ (1:100), anti-Myc (1:100) or anti-B23 (1:250) antibodies followed by three washes in cold PBS and subsequent incubation with goat anti-mouse IgG coupled to Alexa Fluor 488 or goat anti-rabbit IgG coupled to Alexa Fluor 594 (1:500; Thermofisher Scientific Inc.) for 1 h at room temperature. After three washes in cold PBS, cells were incubated with DAPI for 5 min and coverslips were then mounted in ProLong Antifade reagent. Samples were observed at room temperature with a 60× objective under oil immersion and with a numerical aperture (NA) of 1.4 with a Nikon A1 laser scanning confocal microscope (Nikon Canada, Mississauga, Canada).

### RNA immunoprecipitation

C8166-45 cells were lysed in a solution composed of 50 mM Tris–HCl pH 8, 100 mM NaCl, 1 mM EDTA, 1% Triton, and RNase inhibitor. Immunoprecipitation was carried out by adding 100 μl Dynabeads Protein G (Invitrogen) followed by incubation with 10 μg control non-specific IgG or anti-HBZ antibody for 2 h at room temperature. Cell extracts were then incubated with the antibody-bead complex overnight at 4°C. After three washes with cold PBS-Tween (2%), beads were treated with 1 ml TRIzol followed by DNase (2 U) treatment for 10 min at 37°C (Biobasic Inc.). RT was next performed on 2 μg RNA in the presence of High Reverse Transcriptase (Biobasic Inc) and oligo(dT)18 for 2 h at 37°C (Thermofisher Scientific Inc.). After cDNA synthesis, PCR amplification was conducted with the Vent DNA Polymerase (New England Biolabs, Whitby, Canada) in the presence of the following forward/reverse primer pairs: 5′-AACTGTCTAGTATAGCCATC-3′ (primer 21–5) and 5′-CGCAGAGTTGAACAAGCAGG-3′ (primer 20–19); 5′-CAGGCAAGCATCGAAACAGC-3′ (primer Exon-1-3) and primer 20–19 (Cavanagh et al., 2006). PCR products were run on a 1.5% agarose gel and resulting amplicons were sequenced.

### Anchorage-independent growth assay

HeLa cells (1.2 × 10^6^/well) were transfected with pEGFP-C1, sHBZ, uHBZ and/or DsRed-B23 expressing vectors (total of 2 μg DNA/well). After 24 h, for each transfection condition, cells were counted, and 100 cells were mixed in 0.3% agar-1× DMEM medium solution and seeded in each well of a 6 well-plate precoated with 0.6% agar-1× medium solution. Cells were covered with 0.5 ml of medium, which was replenished every 3 days. At 15-day post-transfection, plates were read and analyzed with a GloMax plate reader.

### Cell viability assay

MT2 and C8166-45 cells (1 × 10^6^) were transfected with 50 nM siNC, or NPM1/B23-specific siRNA-21 and siRNA-22 using the Neon® Transfection System and transfection kit (Thermofisher Scientific Inc., MPK5000 and MPK1096). Cells were next seeded into triplicate wells on 96-well plate. After 48 h, 10 μl Cell counting KIT-8 buffer (CCK-8, Sigma-Aldrich) was added into each well and incubated for 4 h at 37°C. Absorbance was measured at 450 nm with the EnSpire 2,300 Multilabel Reader (PerkinElmer) at room temperature.

### Statistical analysis

All statistical analyses were conducted with the Graphpad Prism 9. Comparisons between groups were carried out using one-way ANOVA for comparison tests. In all cases, a *p* value less than 0.05 was considered significant.

## Data availability statement

The original contributions presented in the study are included in the article/supplementary material. Further inquiries can be directed to the corresponding authors.

## Ethics statement

The studies involving human participants were reviewed and approved by the Medical Research Ethics Committee of the Second Affiliated Hospital of Fujian Medical University and Fujian Province Government (2016-SAH-FMU-No.48). Written informed consent for participation was not required for this study in accordance with the national legislation and the institutional requirements.

## Author contributions

ZL, ÉL, GL, SC, ER, and BB have designed the research. J-MM, HG, and YZ have provided material or PBMC samples. ZL, ÉL, YXie, YXia, and J-MP have conducted the experiments. ZL, ÉL, YXia, RL, J-MP, and BB have analyzed the data. ZL, ÉL, J-MP, and BB wrote the paper. All authors contributed to the article and approved the submitted version.

## Funding

This work was supported by the National Natural Science Foundation of China (81902076) the National Mega-Project for Infectious Disease (2018ZX10301408), CAMS Innovation Fund for Medical Sciences (2018-I2M-3-004), Startup Fund for scientific research, and the Health and Education Joint Research Project of Fujian Province (2019-wj-13).

## Conflict of interest

The authors declare that the research was conducted in the absence of any commercial or financial relationships that could be construed as a potential conflict of interest.

## Publisher’s note

All claims expressed in this article are solely those of the authors and do not necessarily represent those of their affiliated organizations, or those of the publisher, the editors and the reviewers. Any product that may be evaluated in this article, or claim that may be made by its manufacturer, is not guaranteed or endorsed by the publisher.

## Supplementary material

SUPPLEMENTARY Figure S1 Co-localization of NPM1/B23 and HBZ in COS-7 cells. COS-7 cells were co-transfected with expression vectors for GFP-fused uHBZ or sHBZ and DsRed-B23. Cells were analyzed by confocal microscopy for HBZ and NPM1/B23.

SUPPLEMENTARY Figure S2 Absence of HBZ-specific signals in immunoprecipitation experiments in control T cells. Extracts from 293T cells transfected with pMyc-sHBZ and from C8166-45 were immunoprecipitated with anti-B23 or control IgG antibodies. Immunoprecipitated and total extracts were analyzed by Western blot with anti-HBZ or anti-B23 antibodies.
