# Supplementary figures and images for: A newly identified interaction between nucleolar NPM1/B23 and the HTLV-I basic leucine zipper factor in HTLV-1 infected cells
Source: Front Microbiol. 2022 Dec 1;13:988944. doi: 10.3389/fmicb.2022.988944 (PMC9753777; doi:10.3389/fmicb.2022.988944)

Figure S1

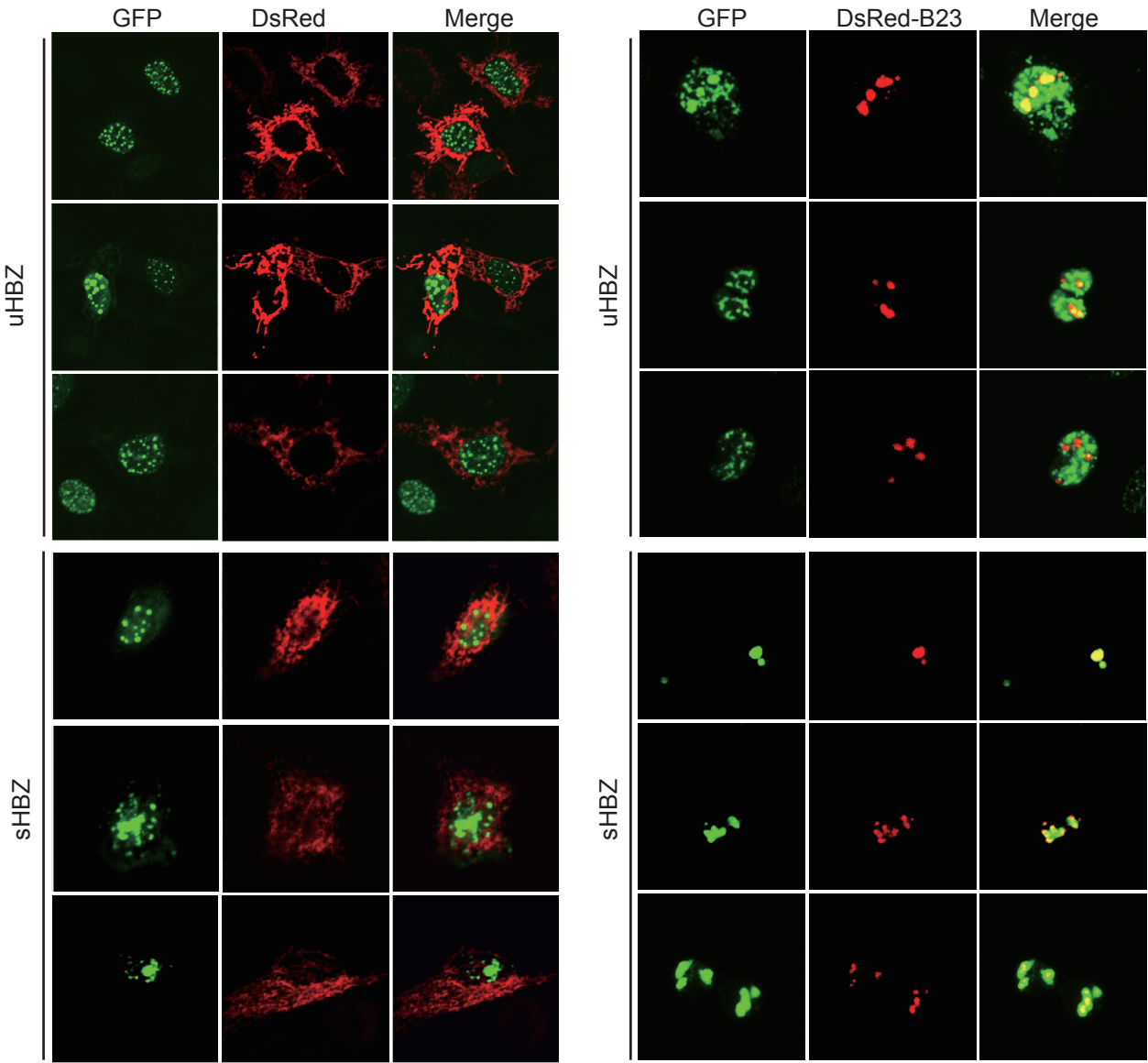

Supplement: SUPPLEMENTARY Figure S1 — Co-localization of NPM1/B23 and HBZ in COS-7 cells. COS-7 cells were co-transfected with expression vectors for GFP-fused uHBZ or sHBZ and DsRed-B23. Cells were analyzed by confocal microscopy for HBZ and NPM1/B23. [file Image_1.pdf]

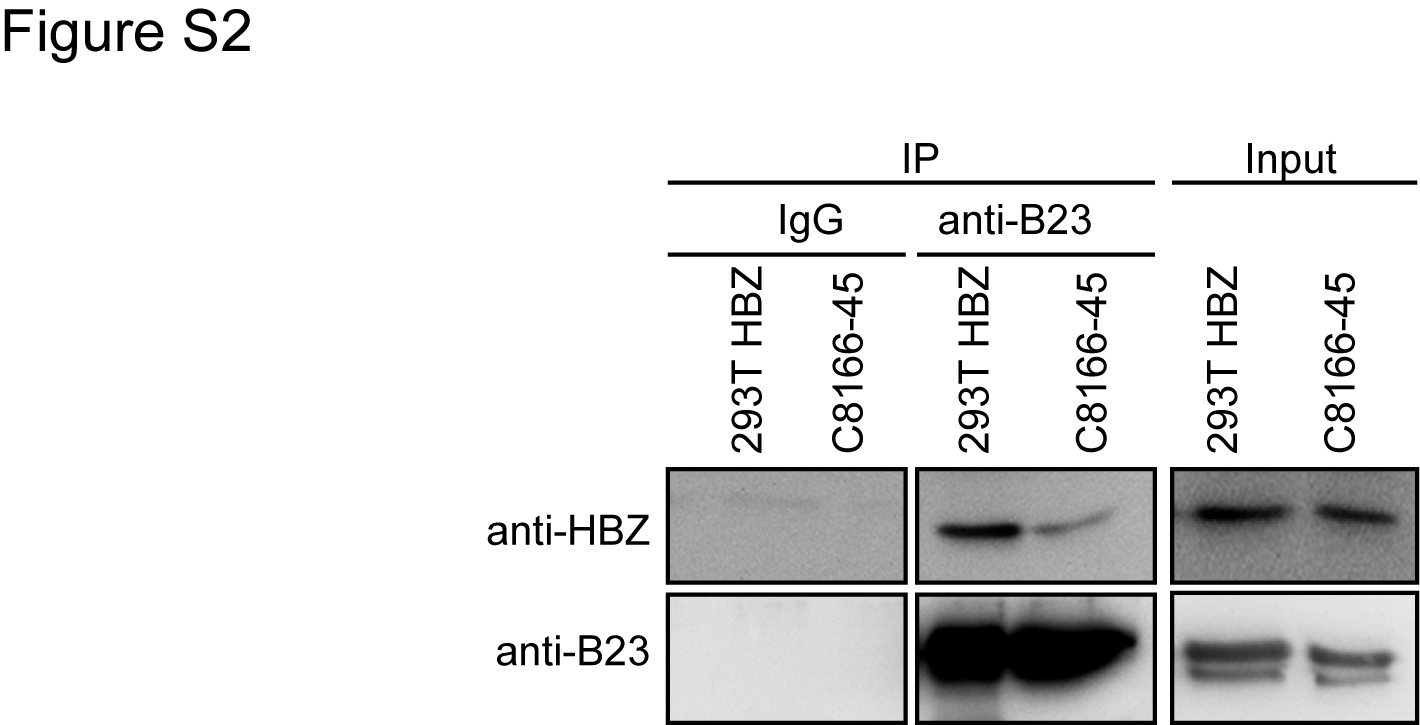

Supplement: SUPPLEMENTARY Figure S2 — Absence of HBZ-specific signals in immunoprecipitation experiments in control T cells. Extracts from 293T cells transfected with pMyc-sHBZ and from C8166-45 were immunoprecipitated with anti-B23 or control IgG antibodies. Immunoprecipitated and total extracts were analyzed by Western blot with anti-HBZ or anti-B23 antibodies. [file Image_2.tif]
